# Effect of Chinese herbal medicine on sexual characteristics of females with precocious puberty: a meta-analysis

**DOI:** 10.3389/fphar.2025.1703077

**Published:** 2025-11-10

**Authors:** Ming Wang, Rui Zhao, Jing Wang

**Affiliations:** Department of TCM, Hainan Women and Children’s Medical Center, Haikou, Hainan, China

**Keywords:** sexual characteristics, meta-analysis, traditional Chinese medicine, Chinese herbal medicine, systematic review

## Abstract

**Background:**

Precocious puberty (PP) in girls is a growing concern globally, characterized by the early onset of sexual characteristics. Chinese Herbal Medicine (CHM) has been proposed as a therapeutic intervention. This meta-analysis evaluates the effects of CHM on primary and secondary sexual characteristics in females with precocious puberty.

**Methods:**

A comprehensive literature search was conducted in Medline, Web of Science, Scopus, China National Knowledge Infrastructure Chinese Biomedical Literature Database (CBM), and Wan-fang database for clinical studies evaluating CHM in girls with precocious puberty. Key outcomes analyzed were primary and secondary sexual traits, including the uterine and ovarian volume and breast nucleus diameter. Data was pooled using a random-effects model and expressed as Standardized Mean Differences (SMD) with 95% confidence intervals.

**Results:**

Twenty-five studies involving 2096 patients were included. CHM significantly decreased breast nucleus diameter (SMD = −1.48, 95% CI [−2.04, −0.92]), reduced uterine volume (SMD = −0.75, 95% CI [−1.24, −0.27]), ovarian volume (SMD = −0.63, 95% CI [−0.97, −0.30]), and follicular volume (SMD = −0.87, 95% CI [-1.49, −0.26]). Subgroup analysis showed the integration of CHM and GnRHa resulted in a notable decrease in the development of sexual traits when compared to GnRHa alone. There was no significant difference between CHM and GnRHa in treating sexual traits. Sensitivity analysis was conducted to address the source of heterogeneity.

**Conclusion:**

CHM appears to effectively modulate primary sexual characteristics in females with precocious puberty. The findings of this study suggest that CHM is a good choice for adjuvant therapy along with GnRHa and also may serve as a complementary treatment for patients with PP.

## 1 Introduction

Precocious puberty is a commonly observed pediatric endocrine disorder, defined by the early onset of secondary sexual traits prior to the ages of 8 and 9 in females and males, respectively (Micangeli et al., 2023). The rising prevalence of central precocious puberty has raised societal concerns, linked to changes in lifestyle, dietary habits, and the surrounding social environment (Kang et al., 2023). This condition affects approximately one in every 5,000 children, with a ratio of ten females to one male (Heo et al., 2016). Factors such as excess adipose tissue, exposure to sex hormones, environmental influences, and various congenital disorders can contribute to its development. A thorough medical history and physical examination are essential for the early identification of pathological precocious puberty, which may involve central nervous system abnormalities, adrenal disorders, gonadal tumors, brain tumors, and genetic anomalies (Mucci and Clemente, 2022; Ismail et al., 2024).

The initiation of puberty is marked by the emergence of a breast bud (breast stage 2) in females and a testicular volume of 3–4 mL (genital stage 2) in males, both of which are triggered by the central activation of the HPG axis (Guidi and Sapra, 2025). The cessation of the inhibition of GnRH neurons during puberty leads to its release from the hypothalamus. This pulsatile release of GnRH subsequently prompts the anterior pituitary to secrete LH and FSH, which exert endocrine effects on the gonads, enhancing the production of estrogen or testosterone, via LH, and facilitating gamete development (via FSH), thereby stimulating growth in the target tissues (Hu et al., 2022; Constantin, 2022).

For millennia, practitioners of Traditional Chinese Medicine (TCM) have utilized herbal remedies, as Chinese herbal medicine (CHM), to address a variety of pediatric ailments (Lai et al., 2024; Zou et al., 2023). However, historical texts provide limited insights into the treatment of precocious puberty, likely due to its relatively low prevalence compared to more common conditions such as infectious, respiratory, and gastrointestinal diseases (Zou et al., 2023). Exploring the strategies employed by TCM practitioners in managing PP and assessing the potential effectiveness of TCM in this regard offers a compelling area for research. Nonetheless, there is a significant lack of extensive studies examining TCM interventions for PP (Bai et al., 2020; Chen et al., 2023). In recent years, TCM has emerged as a frequently used therapeutic modality for treating precocious puberty. TCM posits that the root cause of this condition lies in a deficiency of kidney yin, which in turn impacts liver yin. Recently, there has been a marked increase in both animal and clinical research focused on the effectiveness of CHM and other TCM methodes in managing central precocious puberty (CPP) (Lin et al., 2017; Ma et al., 2023).

The rising prevalence of PP has escalated concerns beyond its epidemiological footprint to encompass significant psychological and social consequences for affected children. Early onset of secondary sexual characteristics is linked to increased risks of anxiety, depression, and social maladjustment due to the discordance between physical and emotional development and peer maturation (Mendle et al., 2007). These adverse impacts underscore the urgency of effective interventions, as untreated PP not only predispose patients to long-term health issues such as metabolic syndrome and compromised adult height but also increase the risk of psychological and social distress in involved individuals.

This meta-analysis aims to determine whether CHM can effectively delay or mitigate the onset of sexual characteristics. Specifically, this study aims to quantify the impact of CHM on breast nucleus diameter, uterine volume, ovarian volume, and follicular development, assess the potential role of CHM as an adjuvant or complementary treatment for precocious puberty by comparing the therapeutic efficacy of CHM alone or the combination of CHM and GnRHa versus GnRHa alone, and also determine and compare the efficacy of various Chinese herbal formulas.

## 2 Materials and methods

We adhered to the ConPhyMP consensus reporting guidelines and completed the ConPhyMP checklist for preparations (Heinrich et al., 2022). For every included polyherbal mixture, we extracted the available details, including common name (used in Chinese herbal medicine), ingredients with the scientific names, which are summarized in the Results Table 1, and provided the ConPhyMP checklist in the Supplementary File S1. Items missing from primary reports are explicitly noted as “not reported.” All botanical names reported in this review were taxonomically verified against *Plants of the World Online* (Royal Botanic Gardens, Kew) (results available in Table 1).

**TABLE 1:** Characteristics of herbal formula used in the included studies.

Study	Mean age-Intervention	Mean age-Control	Total sample size	Sample size intervention	Sample size control	Duration of treatment	Treatment in intervention group	Treatment in control group	Type of GnRHa
Depei,C,1998	_	_	86	51	35	30 months (mean)	Combination of CHM and GnRHa	GnRHa	Leuprorelin
Chen,2010	6.86 ± 0.78	6.72 ± 0.83	100	50	50	6 monh	Zaoshu 3 formula	GnRHa	Medroxyprogesterone
Ma,2011	5.9∼8.1	5.9∼8.1	88	48	40	24 months	GnRHa + Zhibai dihuang	GnRHa	Triptorelin
Gu,2013	6.9 ± 1.6	6.9 ± 1.6	56	28	28	_	GnRHa + Zhibai dihuang	GnRHa	Triptorelin
Fu,2013	7. ± 1.8	7. ± 1.8	102	51	51	36 months	GnRHa + Zhibai dihuang	GnRHa	Triptorelin
Xu,2015	7.32 ± 1.28	7.40 ± 1.31	140	70	70	12 months	GnRHa + Zaoshu formula	GnRHa	Triptorelin
Zhu,2015	_	_	120	60	60	_	GnRHa + Zhi Bai Di Huang Wan	GnRHa	Triptorelin
Cui Yan,2015	6.9 ± 1.7	6.9 ± 1.7	51	27	24	6 monh	Zhi Bai Di Huang Tang	GnRHa	Triptorelin
Qi,2016	6.80 ± 0.32	7.10 ± 0.45	72	36	36	18 months	GnRHa + Dabuyin pill	GnRHa	Triptorelin
Zhang,2016	6.78 ± 0.12	6.79 ± 0.11	70	35	35	6 monh	GnRHa + Dabuyin pill	GnRHa	Triptorelin
Gan DM,2016	8.11 ± 1.97	8.02 ± 2.01	83	45	38	6 months	GnRHa + Dabuyin pill	GnRHa	Leuprorelin
Chen YZ,2018	7.13 ± 0.78	7.24 ± 0.66	64	32	32	6 months	Ziyin xiehuo formula	GnRHa	Triptorelin
Shen YX,2018	7.64 ± 2.68	7.51 ± 2.64	88	44	44	6 months	GnRHa + Zhibai dihuang	GnRHa	Triptorelin
Ying,2019	7.01 ± 0.48	7.17 ± 0.69	80	40	40	6 months	Zhibai jianghuo decoction	GnRHa	Leuprorelin
Chou,2020	7.08 ± 0.42	7.19 ± 0.48	104	52	52	12 months	GnRHa + Zhibai dihuang	GnRHa	Triptorelin
Liu,2020	8.12 ± 1.03	8.03 ± 1.12	80	40	40	3 months	GnRHa + Zhibai dihuang + Dabuyin pill	GnRHa	Triptorelin
Li,2021	6.31 ± 1.20	6.35 ± 1.22	60	30	30	6 months	Zhibai jianghuo decoction	GnRHa	Triptorelin
Zhao,2021	7.15 ± 1.08	7.15 ± 1.29	98	49	49	6 months	GnRHa + Zhibai dihuang	GnRHa	Triptorelin
Huai,2021	7.5 ± 1.4	7.2 ± 1.1	100	50	50	6 months	GnRHa + Zhibai dihuang	GnRHa	Triptorelin
Zhao 2021	6.41 ± 0.40	6.38 ± 0.36	80	40	40	3 months	GnRHa + Dabuyin pill	GnRHa	Leuprorelin
Jiang,2022	8.05 ± 0.56	8.37 ± 0.39	40	20	20	3 months	Zhibai dihuang decoction	GnRHa	Leuprorelin
Huang,2022	6.85 ± 0.29	6.37 ± 0.28	62	31	31	3 months	GnRHa + Zhibai dihuang	GnRHa	Triptorelin
Gong HT,2022	6.44 ± 1.29	6.51 ± 1.37	81	40	41	4months	GnRHa + Zhibai jianghuo	GnRHa	Triptorelin
Wu,2023	8.18 ± 0.68	8.26 ± 0.57	106	53	53	6 months	GnRHa + Zhibai dihuang	GnRHa	Leuprorelin
Lu,2023	7.04 ± 1.24	6.84 ± 1.17	85	43	43	3 months	GnRHa + Ziyin xiehuo formula	GnRHa	Leuprorelin

### 2.1 Search strategy

We searched Medline, Web of Science, and Scopus, China National Knowledge Infrastructure (CNKI), Chinese Biomedical Literature Database (CBM), and Wan-fang database using keywords related to TCM, precocious puberty, including Precocious Puberty, Premature Puberty. Early Puberty, Sexual Precocity, Precocities, Chinese herbal medicine, Chinese herbal preparations, Herbal supplements, Chinese herbal formula, Chinese herbal remedy, and Chinese traditional medicine, Traditional medicine. Then, clinical trials investigating the effect of Chinese herbal formula used in traditional Chinese medicine in female patients with PP were considered.

### 2.2 Study selection and data extraction

To ensure the relevance and appropriateness of the articles, we conducted a meticulous screening process based on their titles and abstracts. Two authors independently screened the included studies after searches in databases by reviewing titles and abstracts; any discrepancies between the 2 authors were resolved by a third author. Any studies that were deemed irrelevant to our research were excluded. Based on this examination, we further classified them as either included or excluded. All full-text manuscripts were thoroughly evaluated to determine their eligibility, considering factors such as being written in English or Chinese and involving human subjects. Studies that utilized animal models, reviews, and congress abstracts, or were written in languages other than English or Chinese, were deliberately excluded from our analysis. Two independent reviewers extracted demographic and clinical data, and any discrepancies between the 2 reviewers were resolved by a third reviewer. In situations where additional information or clarification was required, we made efforts to contact the corresponding author. Inclusion criteria applied for studies were:

- Clinical trials involving female patients with PP.
- Interventions using any sort of CHM, whether decoction, pill, powder, etc., alone or combined with Western therapy.
- Outcomes assessing primary or secondary sexual characteristics (e.g., breast development, uterine/ovarian volume).

The items of exclusion criteria were:

- Animal or *in vitro* studies.
- Reviews, case reports, and non-comparative studies.

### 2.3 Statistical analysis

The statistical analysis was performed utilizing Stata (version 17) and RevMan 5.1 software. To address potential variability among the studies, a random-effects model was applied to compute the Standardized Mean Difference (SMD). The I^2^ statistic served as a measure of heterogeneity across the studies, with thresholds of 25%, 50%, and 75% denoting low, moderate, and high levels of heterogeneity, respectively. Sensitivity analysis utilizing Leave-one-out analysis was conducted to identify the sources of heterogeneity.

### 2.4 Quality assessment

The assessment of the studies included in the analysis was performed. Study quality was evaluated using the Cochrane Risk of Bias Assessment Tool, version 2 (ROB2), a valid tool for assessing risk of bias that evaluates items including random sequence creation, allocation concealment, blinding techniques, inadequate data, selective outcome reporting, and other biases. Two reviewers independently scored the included studies based on the mentioned criteria. Any disagreements between the two researchers were addressed through discussion and consultation by a third author.

## 3 Results

### 3.1 Study characteristics

**FIGURE 1:**
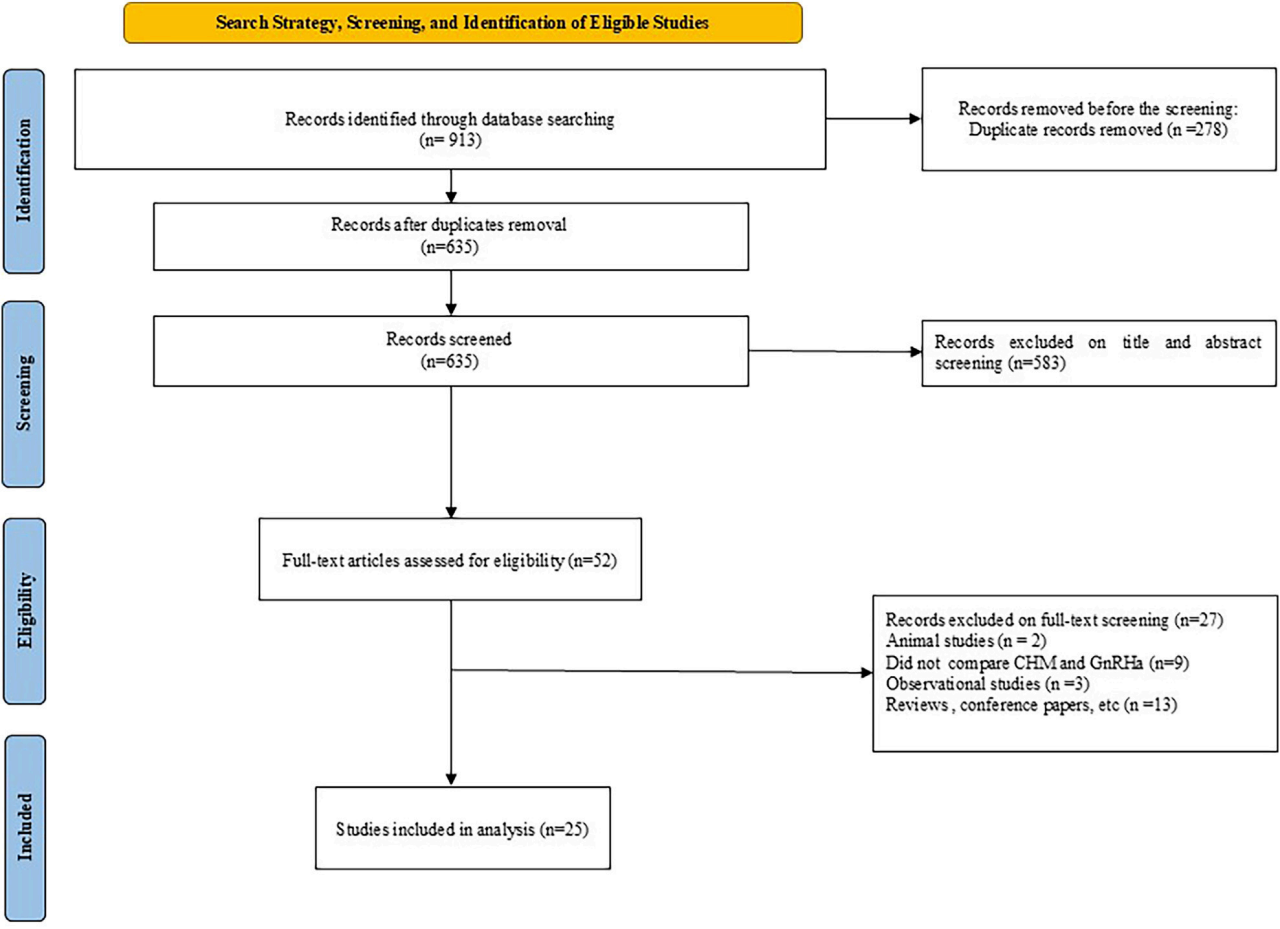
PRISMA diagram of the selection process.

The process of screening the literature resulted in a total of 913 studies. After eliminating duplicate entries using Endnote X9.3.3, 635 studies remained for evaluation. A comprehensive review of titles and abstracts led to the identification of 52 studies as appropriate for inclusion. Following an in-depth assessment of the full texts, 25 studies (Heinrich et al., 2022; Depei et al., 1998; Chen, 2010; MA, 2011; FU, 2013; G and U, 2013; CUI et al., 2015; Xu and Jiang, 2015; Gan, 2016; Qi et al., 2016; Zhang, 2016; Chen, 2018; Shen, 2018; Ying, 2019; Chou and Wu, 2020; Liu and Jin, 2020; Huai et al., 2021; Li, 2021; Zhao et al., 2021; Z et al., 2021; Gong, 2022; Huang and Li, 2022; Jiang, 2022; Lu, 2023; Wu et al., 2023; Zhu, 2015) were selected. The selection process is depicted in Figure 1. These studies were published between 1998 and 2023. In total, 2096 patients were involved across the studies, with 1,065 in the control group and 1,032 in the experimental group. The duration of the interventions varied from 3 to 30 months, and the fundamental characteristics of the 25 studies included are summarized in Table 2. The formula most frequently prescribed in the studies reviewed was Zhibai dihuang, followed by Dabuyin, Ziyin xiehuo formula, Zhibai jianghuo, and the combination of Zhibai dihuang with Dabuyin pill. The detailed information of the herbal formula (ingredients and scientific names) used in the included studies are available in Table 1.

**TABLE 2:** Summary of the fundamental characteristics of the included studies and patients.

Name of CHM	Formula	Ingredients	Taxonomically status
Zhibai Dihuang	Herbal formula	Rehmannia glutinosa, Dioscorea oppositifolia, Cornus officinalis, Paeonia suffruticosa, Alisma plantago-aquatica, Poria cocos, Anemarrhena asphodeloides, Phellodendron amurense	Poria cocos was not found in the search database All other ingredients were accepted
Dabuyin Wan	Herbal formula	Anemarrhena asphodeloides, Phellodendron chinense, Rehmannia glutinosa, Chinemys reevesii	Chinemys reevesii was not found in the search database All other ingredients were accepted
Zao shu pi	Single herb	Ziziphus jujuba	Accepted
Ziyin Jiang Huo Tang	Herbal formula	Angelica sinensis, Paeonia lactiflora, Rehmannia glutinosa, Asparagus cochinchinensis, Ophiopogon japonicus, Atractylodes macrocephala, Citrus reticulata, Glycyrrhiza uralensis, Rehmannia glutinosa, Anemarrhena asphodeloides, Phellodendron chinense, Zingiber officinale, Ziziphus jujuba	Zingiber officinale Was not found in the search database All other ingredients were accepted
Ziyin Xiehuo	Herbal formula	Anemarrhena asphodeloides, Phellodendron chinense	Ingredients were accepted
Zhibai jianghuo	Herbal formula	Anemarrhena asphodeloides, Phellodendron chinense, Rehmannia glutinosa, Cornus officinalis, Dioscorea species, Poria cocos, Paeonia suffruticosa, Alisma plantago-aquatica	Poria cocos was not found in the search database All other ingredients were accepted

### 3.2 Sexual characteristics

#### 3.2.1 Uterine volume

**FIGURE 2:**
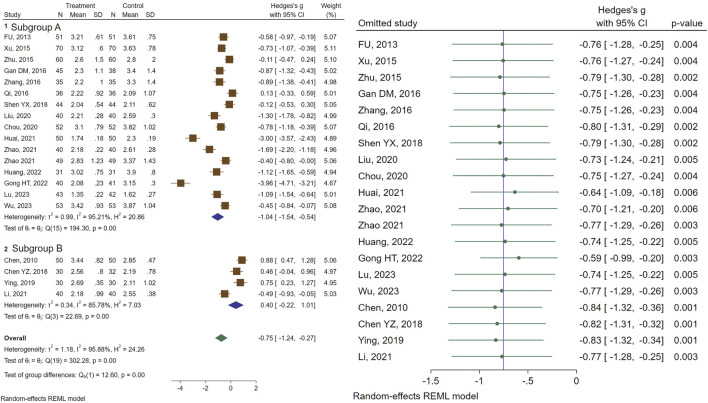
Forest plot of the efficacy of CHM in reducing UV (right) and the sensitivity analysis to address studies causing heterogeneity (left).

Of the included studies, 20 assessed the efficacy of CHM on uterine volume (UV). Our analysis demonstrated a significant size reduction in UV was recorded in patients receiving CHM (SMD = −0.75, 95% CI [−1.24, −0.27], I^2^ = 95%). Subgroup analysis revealed a significant reduction in UV (SMD = −1.04, 95% CI [−1.54, −0.54], I2 = 95%) for the GnRHa + CHM receiving patients as compared to control (GnRHa-recieving) group, whereas no significant changes in UV (SMD = 0.40, 95% CI [−0.22, 1.01], I2 = 85%) were noted for the CHM in comparison with the GnRHa (Figure 2). Further subgroup analysis was performed to address the source of remaining heterogeneity (Figure 2).

#### 3.2.2 Mean ovarian volume

**FIGURE 3:**
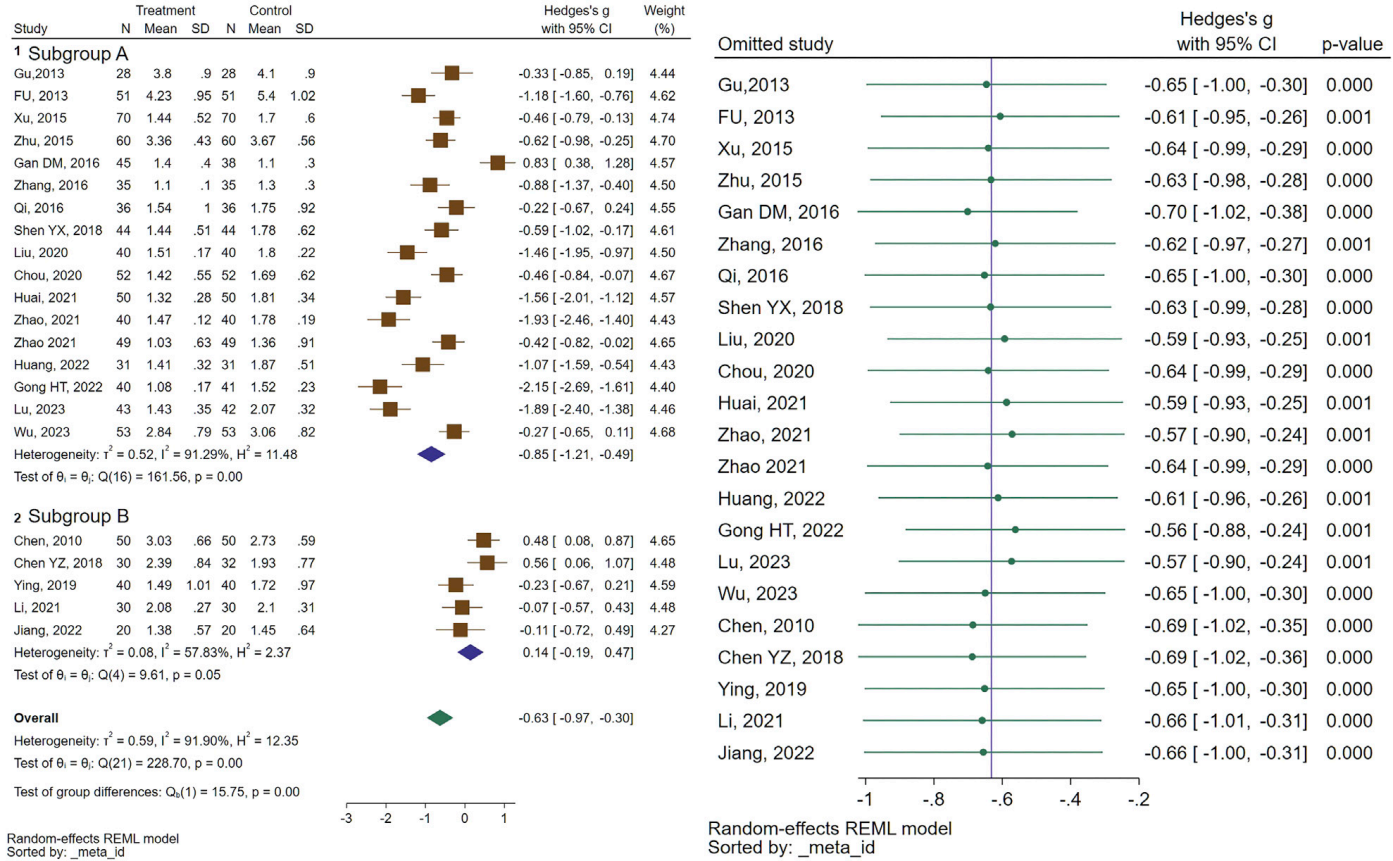
Forest plot of the efficacy of CHM in reducing MOV (right) and the sensitivity analysis to address studies causing heterogeneity (left).

In total, 22 studies assessed the efficacy of CHM formulas on mean ovarian volume (MOV). Regarding MOV, a significant reduction in size was recorded (SMD = −0.63, 95% CI [−0.97, −0.30], I^2^ = 91%). Subgroup analysis indicated a significant decrease in MOV size for the combination therapy of CHM and GnRHa, while no significant change was observed for the CHM treated patients compared to the control group (treated with GnRHa) (SMD = −0.85, 95% CI [−1.21, −0.49], I2 = 91%, and SMD = 0.14, 95% CI [−0.19, 0.47], I2 = 57%, respectively). The forest plot and sensitivity analysis on MOV are illustrated in Figure 3.

#### 3.2.3 Follicular volume

**FIGURE 4:**
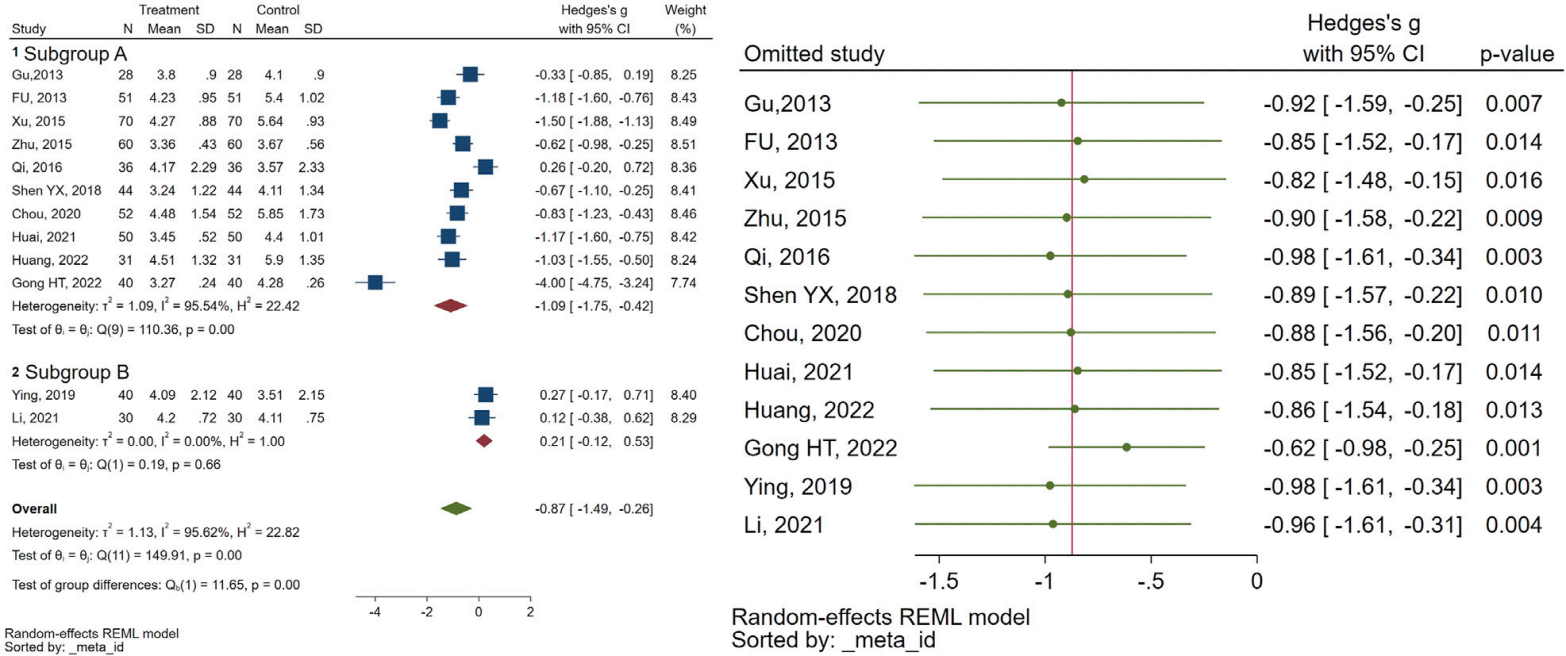
Forest plot of the efficacy of CHM in reducing FV (right) and the sensitivity analysis to address studies causing heterogeneity (left).

Twelve randomized controlled trials examined follicular volume (FV). The meta-analysis revealed a notable reduction in FV (SMD = −0.87, 95% CI [-1.49, −0.26], I2 = 95%), in the individuals receiving CHM compared to the control group. Subgroup analysis indicated that while the combination of GnRHa and CHM was significantly more effective than GnRHa for FV (SMD = −1.09, 95% CI [-1.75, −0.42], I2 = 95%), there was no significant difference between CHM and GnRHa for FV (SMD = 0.21, 95% CI [-0.12, 0.53], I2 = 0%). Sensitivity analysis was performed to find the source of heterogeneity. Figure 4 illustrates the data concerning the use of CHM for FV.

#### 3.2.4 Breast nucleus diameter

**FIGURE 5:**
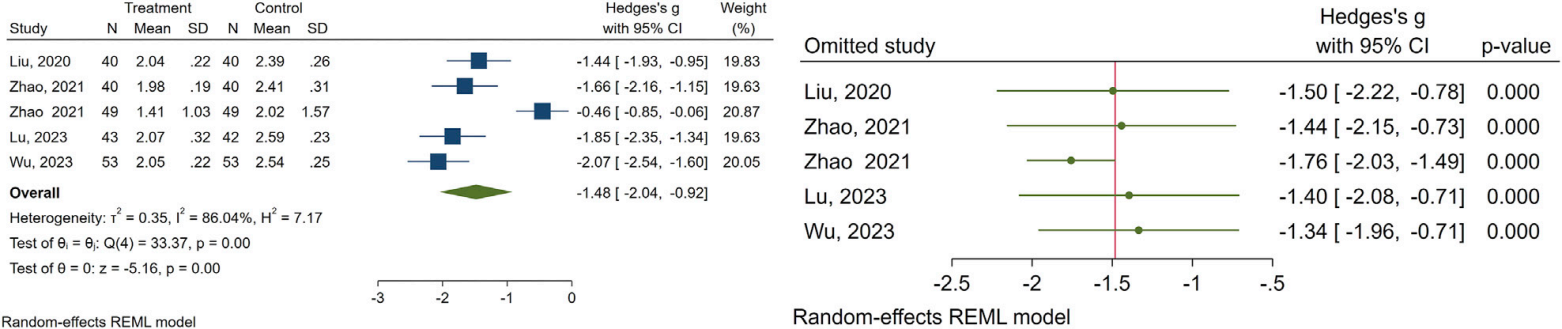
Forest plot of the efficacy of CHM in reducing breast nucleus diameter (right) and the sensitivity analysis to address studies causing heterogeneity (left).

A total of 5 studies evaluated the effectiveness of CHM on breast nucleus diameter. The meta-analysis results indicated that the experimental group exhibited notable enhancements in breast nucleus diameter (SMD = −1.48, 95% CI [−2.04, −0.92], I2 = 86%) in comparison to the control group. To address studies causing the heterogeneity, we performed a sensitivity analysis. The data pertaining to the breast nucleus diameter is illustrated in Figure 5.

#### 3.2.5 Effect of different heral formula

Different herbal formulas can have different effects on the development of sexual characteristics. To identify the source of heterogeneity and assess the impact of each herbal formula, we conducted the analysis based on the herbal formula. Our analysis revealed that the combination of GnRHa with Zhibai dihuang and Dabuyin, in comparison with the GnRHa alone, was significantly linked to the delay of sexual characteristics improvements, whereas Zhibai jianghuo decoction showed no such association. Table 3 presents the effects of each herbal formula along with the corresponding heterogeneity.

**TABLE 3:** Effects of different herbal formulas on sexual characteristics and their corresponding heterogeneity.

CHM formula	Sexual trait	SMD with CI	I2	P-value for heterogenity
GnRHa + Zhibai dihuang	Breast Nucleus	−1.87 (−2.27, −1.47)	26.33%	0.24
Zhibai jianghuo decoction	Follicular volume	0.21 (−0.12–0.53)	0%	0.66
GnRHa + Zhibai dihuang	Follicular volume	−0.88 (−1.13, −0.63)	46%	0.09
Zhibai jianghuo decoction	Mean Ovarian Volume	−0.16 (−0.49, 0.17)	0%	0.63
GnRHa + Zhibai dihuang	Mean Ovarian Volume	−1.04 (−1.43, −0.66)	87.6%	<0.001
GnRHa + Dabuyin pill	Mean Ovarian Volume	−0.09 (−1.06, 0.89)	92.5%	<0.001
Zhibai jianghuo decoction	Uterine volume	0.12 (−1.00,1.34)	92.18%	<0.001
GnRHa + Zhibai dihuang	Uterine volume	−0.97 (−1.63, −0.31)	94.68%	<0.001
GnRHa + Dabuyin pill	Uterine volume	−0.51 (-0.97–0.04)	77.23%	0.01

### 3.3 Quality assessment

**FIGURE 6:**
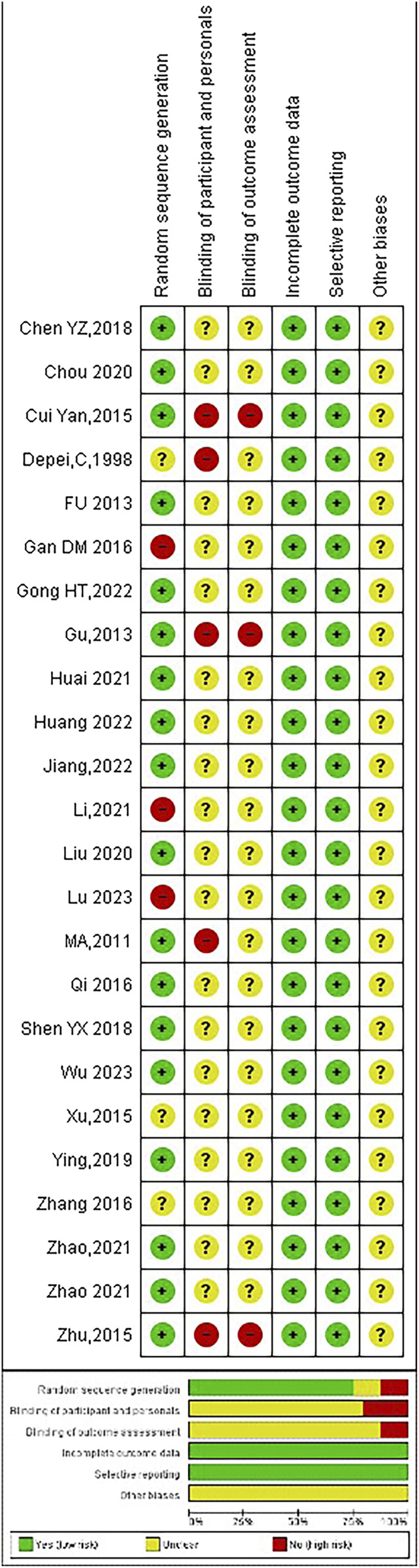
Summary of risk of bias assessment.

The outcomes of the risk-of-bias assessment conducted on the included studies are indicated in Figure 6.

## 4 Discussion

This study aims to investigate the effect of Chinese herbal medicine on the sexual characteristics of females with precocious puberty in the available literature. The study included 25 studies with a total of 2096 patients. Our findings demonstrate that CHM can effectively delay the development of sexual characteristics in girls with PP. The herbal formulations used in these studies appear to suppress the HPG axis by modulating GnRH and kisspeptin signaling.

While no prior meta-analysis focused precisely on these specific parameters in a pooled manner as ours does, our results align well with earlier clinical investigations highlighting CHM’s potential to optimize conventional therapies. The findings from this meta-analysis contribute meaningfully to the growing body of evidence supporting CHM as an adjunctive, or alternative in some cases, interventions in managing pp in females, particularly in mitigating the advancement of primary and secondary sexual characteristics. Although previous meta-analyses and clinical studies on CHM in precocious puberty are relatively sparse, a recent systematic review and meta-analysis by Cheng et al. (Cheng and Wan, 2025) reported similar findings whereby CHM formulations were shown to be effective when combined *w*th Western medicine, as compared to Western medicine alone. The integration of CHM with GnRHa treatment demonstrated an enhanced reduction in sexual characteristic development compared to GnRHa alone, extending prior observations from clinical trials that suggested adjunctive CHM improves outcomes such as hormone profile regulation and skeletal maturation delay.

Further supporting our findings, Lee et al. (Lee et al., 2018) conducted a meta-analysis on 9 RCTs focusing on herbal medicine for idiopathic PP, reporting significant reductions in serum LH and E2 levels, bone age, and UV in patients receiving herbal medicine compared to controls. Their findings highlight similar efficacy with a favorable safety profile, emphasizing herbal medicine as a potential alternative or complementary approach to standard GnRHa therapy, particularly given concerns about growth rate and final height limitations with long-term GnRHa use.

Moreover, the integration of CHM, as a routine method of TCM with Western medicine (WM) resulted in a notable decrease in the development of sexual traits when compared to WM alone. There was no significant difference between CHM and WM in treating sexual traits, indicating that CHM may serve as a complementary treatment for patients with PP, considering that the monthly expense for GnRH analogs is approximately $150, whereas the cost for 9 g of concentrated TCM herbal granules daily is about $15 per month. Consequently, CHM may present a cost-effective alternative with fewer adverse effects for the treatment of patients with PP.

TCM lacks a distinct terminology or classification for the condition known as ‘precocious puberty’. Based on TCM principles, the underlying pathogenesis of central precocious puberty is attributed to deficiencies in kidney yin, an imbalance between yin and yang, depletion of yin, excessive fire, and the early activation of Tiangui. (Fuqua, 2013). The Rehmannia Pill, also referred to as Zhi-Bai-Di-Huang-Wan, alongside Phellodendron and Anemar, emerged as the most commonly prescribed herbal treatment for idiopathic precocious puberty in the reviewed literature. This formulation comprises eight distinct herbs: Shou-Di (Rehmanniae Radix Praeparata), Zhi-Mu (Anemarrhenae Rhizoma), Shan-Yao (Dioscoreae Rhizoma), Huang-Bai (Phellodendri Cortex), Ze-Xie (Alismatis Rhizoma), Shan-Zhu-Yu (Corni Fructus), Fu-Ling (Poria), and Mu-Dan-Pi (Moutan Cortex). The principles of TCM indicate that Zhi-Bai-Di-Huang-Wan is capable of nourishing yin and dispelling fire, akin to the effects observed with Da-Bu-Yin-Wan (Chen and Chen, 2009). The latter has been shown to reduce the hypothalamic-pituitary-gonadal axis by lowering Kiss-1/GPR54 expression and inhibiting GnRH production in the hypothalamus of animal models. Furthermore, TCM herbal combinations have been noted to influence hypothalamic Kisspeptin expression, downregulate elevated GnRH levels, and significantly delay sexual maturation in animal studies (Chen et al., 2023; Zhang et al., 2018). Various Chinese investigations have also indicated that herbal remedies aimed at nourishing yin and purging fire can affect the hypothalamic-pituitary-ovarian axis, thereby postponing skeletal maturity in individuals with idiopathic precocious puberty. Despite the current lack of extensive research on Zhi-Bai-Di-Huang-Wan, our study suggests that this herbal formula shows potential for the treatment of idiopathic precocious puberty and warrants further investigation in both research and clinical settings (Wang et al., 2014; Zeng et al., 2015). In addition to Xin-Yi-San, Zhi-Bai-Di-Huang-Wan, Xiao-Qing-Long-Tang, Ge-Gen-Tang, and Cang-Er-San Xin-Yi-Qing-Fei-Tang were recognized as part of the ten most frequently prescribed formulas for idiopathic precocious puberty (Lee et al., 2020).

Mai-Ya (Hordei Fructus Germinatus) is the most commonly prescribed single herb, accounting for approximately fifty percent of all prescriptions, aside from herbal formulas (Lien et al., 2016). In the context of TCM, it is regarded as beneficial for stomach harmony and digestive support. However, it is crucial to recognize that excessive consumption of Mai-Ya may lead to adverse effects such as the cessation of lactation and a decrease in breast swelling (Sheng et al., 2018; Yang et al., 2021). Furthermore, breast development is generally the first sign of sexual maturation in females during puberty. Consequently, the administration of Mai-Ya is believed to potentially delay the onset of puberty, particularly regarding breast growth (Alghamdi, 2023).

The theoretical framework of TCM posits that Da-Bu-Yin-Wan has the capacity to nourish yin and eliminate excess heat (NYRF), akin to the properties of Zhi-Bai-Di-Huang-Wan. Herbal formulations exhibiting NYRF effects have been shown to reduce the expression of neurokinin B (NKB) and neurokinin 3 receptor (NK3R) in the hypothalamus, as well as kisspeptin protein levels in the arcuate and periventricular nuclei, and the preoptic area (Koysombat et al., 2023). This downregulation leads to a decrease in gonadotropin-releasing hormone (GnRH) expression, significantly postponing the onset of secondary sexual characteristics in animal models (Lee et al., 2016). Cortex Phellodendri (Huang-Bai) has been identified as a factor that delays puberty by inhibiting GnRH production in the hypothalamus, while simultaneously enhancing growth through increased synthesis and secretion of growth hormone (GH) in the pituitary gland. Zhi-Bai-Di-Huang-Wan is proposed as a promising herbal intervention for precocious puberty, warranting further research and clinical trials. Following this, Jia-Wei-Xiao-Yao-San and Long-Dan-Xie-Gan-Tang rank as the second and third most commonly utilized herbal remedies for precocious puberty, respectively. Jia-Wei-Xiao-Yao-San, a time-honored TCM formula with a legacy spanning over a millennium, aims to rectify liver and spleen blood deficiencies accompanied by heat transformation, often manifesting as gastrointestinal disturbances, mood fluctuations, and menstrual irregularities associated with endocrine dysfunctions. Long-Dan-Xie-Gan-Tang is regarded as effective in addressing precocious puberty syndrome according to TCM principles, particularly in instances characterized by an excess of pathogenic heat in the liver or the downward movement of damp-heat within the liver channel towards the lower Jiao (Lin et al., 2013).

## 5 Limitations and guides for future studies

Our review encountered several limitations. Our findings indicated significant heterogeneity in some of the parameters we assessed. To explore this heterogeneity, we performed subgroup meta-analyses; however, heterogeneity continued to be evident in specific subgroups. This may be attributed to the diverse subtypes employed in each study, as well as the varying effectiveness of each formula or herbal treatment. The type of TCM utilized in the studies may also play a role in this heterogeneity. It is important to note the limitations inherent in the previous meta-analysis and available literature, including variable study designs, small sample sizes, and differences in CHM formula compositions. In the absence of such detailed analyses, our discussion highlights the promising role of CHM while calling for rigorous mechanistic and clinical exploration to solidify its therapeutic place in precocious puberty management. Nonetheless, there is a scarcity of data regarding particular TCM subtypes, highlighting the need for further investigation to overcome this limitation. Another limitation is that some Chinese formulas contain several ingredients. Therefore, the exact effect of each ingredient is not clear. Future studies should also investigate the effect of each ingredient independently and in combination with other ingredients to find the potential synergy. Furthermore, we tried to report the names of herbal ingredients, based on recommendations suggested by Rivera,D. et al. (Rivera et al., 2014), nevertheless, some information was not avilble form included studies. For instance, the proportion and plant part used (leaf, root, fruit). Therefore, future studies should explicitly adhere to accepted guidelines when using herbal mixtures.

To address the limitations highlighted above and better characterize the clinical and psychosocial burden of precocious puberty, future studies should prioritize prospective, longitudinal cohort designs that follow children treated with CHM as adjuvant or alternative therapy from pre-puberty through adolescence and incorporate repeated, standardized assessments of mental health and social functioning as well as physical features and sexual characteristics. Such studies should recruit sufficiently large and socioeconomically and ethnically diverse samples to allow examination of effect modification by sex, socioeconomic status, and ethnicity, and to distinguish biological from contextual drivers. Additionally, to further elucidate the biological mechanisms that link clinical use of CHM in PP timing to subsequent sexual and even psychological outcomes, future basic and translational studies are needed alongside the proposed longitudinal cohort work.

## 6 Conclusion

This meta-analysis supports the efficacy of CHM in delaying primary and secondary sexual development in females with precocious puberty. The integration of CHM with WM resulted in a notable decrease in the development of sexual traits when compared to WM alone. There was no significant difference between CHM and WM in treating sexual traits. Hence, CHM is a good choice for adjuvant therapy along with GnRHa and also may serve as a complementary treatment for patients with PP.

## Data Availability

The original contributions presented in the study are included in the article/supplementary material, further inquiries can be directed to the corresponding author.
